# Based on whole-exome sequencing to explore the rule of Herceptin and TKI resistance in breast cancer patients

**DOI:** 10.1186/s12920-023-01762-x

**Published:** 2024-01-19

**Authors:** Liantao Guo, Hong Cheng, Jianhua Liu, Weikang Shao, Lan Luo, Weijie Zheng, Shengrong Sun, Deguang Kong, Chuang Chen

**Affiliations:** 1 Department of Breast and Thyroid Surgery, Renmin Hospital of Wuhan University, No. 238 Jiefang Road, Wuhan, Hubei 430060 People’s Republic of China; 2 Department of Breast Surgery, The Central Hospital of Enshi Tujia and Miao Autonomous Prefecture, Enshi, Hubei 430060 People’s Republic of China; 3 Department of breast surgery, Hubei Cancer Hospital, Tongji Medical College, Huazhong University of Science and Technology, Hubei Provincial Clinical Research Center for Breast Cancer, Wuhan Clinical Research Center for Breast Cancer, No.116 Zhuo Daoquan South Road, Wuhan, Hubei 430079 People’s Republic of China; 4 Genecast Biotechnology Co., Ltd., Wuxi, Jiangsu 214000 People’s Republic of China; 5 Department of Breast Surgery, The Affiliated Hospital of Guizhou Medical University, No. 28 Guiyi Road, Yunyan District, Guiyang, Guizhou 550001 People’s Republic of China

**Keywords:** Breast cancer, WES, Herceptin resistance, TKI resistance, Immunotherapy

## Abstract

**Background:**

Breast cancer is the second leading cause of cancer-related death in women, and drug resistance during treatment is a major challenge. However, the mechanisms underlying drug resistance are not fully understood. Here we applied whole-exome sequencing (WES) to clarify resistant rules to Herceptin and tyrosine kinase inhibitors (TKIs).

**Methods:**

There are 12 HER2+ breast cancer patients who were done WES. Samples from tumor and surrounding tissues underwent DNA sequencing and analysis. Various experimental and bioinformatics techniques were employed, including genomic capture, mutation analysis (Genome Analysis Toolkit (GATK), etc.), bioinformatics assessments, and drug-gene interaction investigations. Ultimately, the study explored the association of *APOB* gene expression with breast cancer recurrence rates, immune cell infiltration, and drug response.

**Results:**

The C > T mutation frequency was highest in the Herceptin-insensitive (HI) and verification groups, codenamed YI, contrasting with the Herceptin-sensitive (HE) group. No microsatellite instability (MSI)-H patients were in the HE group, but both HI and YI groups had 1 each. Significant differences in transition-transversion (TiTv) were observed in the HI and YI groups rather than the HE group. In the TKI- insensitive (TI) group, C > T mutations were highest, differing from the TKI-sensitive (TE) group. TE group included 2 MSI-H patients. Significant differences in TiTv were found in the TI group rather than the TE group. Mutated *APOB* may resist Herceptin and TKI, increasing immune infiltration. We identified potential drugs targeting it.

**Conclusions:**

Our study suggested that a higher percentage of C > T mutations, significant differences in TiTv, and MSI-H status may indicate Herceptin resistance, while a higher percentage of C > T mutations, significant differences in TiTv, and the absence of MSI-H may indicate TKI resistance in breast cancer patients. For patients resistant to both Herceptin and TKI, mutated *APOB* may play a crucial role in resistance.

**Supplementary Information:**

The online version contains supplementary material available at 10.1186/s12920-023-01762-x.

## Introduction

Over the past few years, there has been a slight increase in the incidence of women’s cancers, particularly breast cancer, which has become the second leading cause of cancer-related deaths among women worldwide [1, 2]. As a result, extensive research has been focused on the treatment of breast cancer. Various treatment options including surgery, radiotherapy, chemotherapy, endocrine therapy, targeted therapy, and immunotherapy have been utilized [3].

Targeted therapy based on the specific subtypes of breast cancer has emerged as a critical approach [3, 4]. HER2-positive breast cancer, accounting for approximately 20–30% of all breast cancer cases, is associated with poor prognosis [5]. Targeting the HER2 pathway has become a significant advancement in breast cancer treatment [6]. Herceptin, a humanized monoclonal antibody targeting the extracellular domain of HER2, was the first approved drug in this category [7–10]. In addition to Herceptin, TKIs have been developed as orally bioavailable drugs targeting HER2 [6]. Examples of TKIs include Lapatinib [11], Neratinib [12, 13], and Tucatinib [14]. However, the response rates to these therapies are relatively low, and acquired resistance is common [15–21]. The underlying mechanisms responsible for this resistance remain unclear. Therefore, it is crucial to identify specific resistance mechanisms and patient subgroups that may be inherently resistant to these therapies before treatment initiation. This knowledge would reduce trial and error approaches, saving time and cost.

Immunotherapy has revolutionized breast cancer management by harnessing the potential of the immune system [22]. Various approaches, including tumor-targeting antibodies, T-cell therapy, vaccines, and immune checkpoint blockade, have been explored [23]. In HER2-positive breast cancer, the level of immune cell infiltration has been associated with immunotherapy efficacy and favorable prognosis. However, identifying patients who do not respond to HER2-targeted therapy but may benefit from immunotherapy remains a challenge. Rapid and accurate patient selection is crucial for timely intervention and improved survival.

Next-generation sequencing technology, particularly WES, enables comprehensive analysis of mutation patterns in cancer [24–26]. WES allows parallel sequencing of numerous genes in a cost-effective and efficient manner [27, 28].

In this study, we employed WES to investigate somatic mutations in breast cancer patients, focusing on understanding the molecular characteristics underlying resistance to Herceptin and TKIs. By integrating WES data with online databases, we identified a novel gene target and potential drugs that could benefit a patient resistant to both Herceptin and TKIs, while demonstrating sensitivity to immunotherapy. This approach holds promise for optimizing therapeutic strategies, enhancing patient outcomes, and advancing personalized medicine in breast cancer management.

## Methods

### Patients

This study enrolled 12 histologically confirmed HER2-positive breast cancer patients. Tumor DNA samples were obtained either through puncture or surgical procedures, while peripheral blood or paracancerous samples served as control DNA samples. In total, 31 samples were collected. The research involving human participants received ethical approval from the Clinical Research Ethics Committee of Renmin Hospital, Wuhan University (WDRY-2022-K228). All patients provided written informed consent for their participation in this study.

### Library preparation

Following the recommended parameters, FFPE DNA ranging from 20 to 500 ng was fragmented to approximately 200 bp fragments using the Covaris M220 system. These DNA fragments were subsequently utilized for library construction with the KAPA Hyper Preparation Kit from KAPA Biosystems (USA). Library quantification was performed using the Qubit dsDNA HS (High Sensitivity) assay kit from Thermo Fisher (USA), while library sizes were determined using the Agilent BioAnalyzer 2100 system from Agilent (USA).

### Targeted region captures and WES sequencing

For targeted region selection, we employed the HyperCap Target Enrichment Kit from Roche (Switzerland). Specifically, we utilized the SeqCap EZ MedExome Target Enrichment kit 384 Reaction panel, following the manufacturer’s instructions for hybridization and washing steps. Subsequently, the captured products were subjected to sequencing using the Illumina Novaseq 6000 instrument with a recommended 150 bp paired end run.

### Mapping

The reads were aligned to the Human Reference Genome hg19 using Burrows-Wheeler Aligner (v0.7.17). Following alignment, the filtered reads were sorted and duplicate flags were applied using the Picard Toolkit (v2.22.0). For local realignment of indels, we employed the GATK (v3.7).

### Single nucleotide variants (SNVs) calling methods

We utilized VarDict for calling SNVs and ANNOVAR for annotation purposes. Compound heterozygous mutations were merged using FreeBayes. To filter SNVs, we applied the following criteria: [1] Sequencing depth < 30 at mutation sites [2]; Exclusion of mutations in the blacklist, intronic regions, or resulting in nonsense mutations [3]; Absence of records in cosmic and snp138 databases [4]; Genes annotated as HLA or NONE [5]; Identification of B mutations based on ljb2_pp2hdiv and ljb2_pp2hvar [6]; Reference to ExAC and gnomAD databases [7]; Lack of support from forward and reverse reads [8]; Non-compliance with frequency and supported reads requirements (except for hotspot sites, where support reads ≥5, plasma frequency ≥ 1, and organization frequency ≥ 5; Hotspot sites filtered according to Medical Department guidelines).

### Enrichment analysis

We conducted gene ontology (GO) enrichment analyses for biological process (BP), molecular function (MF), and cellular component (CC) using the topGO package in the R environment for statistical analysis and visualization. The log2foldchange represents the average mutation frequency of each gene across all samples in the group. We uesd R package ClusterProfiler to perform Kyoto Encyclopedia of Genes and Genomes (KEGG) enrichment analysis [29, 30]. Additionally, we incorporated the Reactome database along with the KEGG pathway.

### Tumor mutational burden (TMB)

We performed a scan for non-equivalent mutations (SNV and Indel) in the coding region and identified driver gene mutations and hotspot mutations from the EXAC/COSMIC database. Subsequently, we selected mutation sites that met specific thresholds for sequencing depth and mutation frequency as candidate sites for tumor mutation load calculation. To calculate TMB, we utilized the candidate sites of mutation load identified above, employing the method of absolute mutation count * 1000000 / total number of exon bases.

### Mutant allele tumor heterogeneity (MATH)

we employed the following methods to filter SNV mutations: [1] SNVs with a frequency greater than or equal to 5 [2]; SNVs with a sequencing depth of at least 50× [3]; Exon region mutations were retained, while synonymous mutations were filtered out [4]; Mutations present at frequencies higher than 10% in breast cancer samples were selected [5]; For sites with frequencies lower than 10% in breast cancer samples, only those with frequencies exceeding 10 times that of breast cancer were retained. We calculated MATH values based on the filtered candidate sites using the following formula: median absolute deviation divided by the median variant allele fraction.

### MSI

We utilized BAM files obtained through mapping and isolated reads containing short tandem repeat (STR) loci. The allele length of each gold marker locus was then calculated. To establish a baseline, we developed self-written modules. The breast cancer samples used in this study were obtained from healthy individuals. Filtering was conducted using the following criteria: sequencing depth > 100×, minimum of 2 reads for allele count, and removal of alleles with a frequency < 5% of the highest frequency. Subsequently, the mean number of alleles and the standard deviation (SD) were calculated. If the number of alleles exceeded the mean number of alleles plus four times the SD of the MSI stable reference values, it was determined to be unstable. The criteria for MSI stability were as follows: unstable locus ≥2 for HIS-H, stable locus ≥4 for HIS-L/MSS, and others categorized as QNS.

### Copy number variation (CNV)

CNVkit (v0.9.2) software was used to analyse the depth distribution of reads on the reference genome, and Somatic CNV was detected for each Tumor and Normal paired sample, then the copy number of all tumour samples was calculated, and finally the CNV status of the samples was determined according to the following conditions: CN > 4 was judged as gain, and CN < 1.2 was judged as loss [31].

### Copy number instability (CNI)

After correction for GC content and length of target region using proprietary algorithms for each region, the read counts were transformed into log2 ratios and converted into Z-score based on Gaussian transformations versus a normal control group (*n* = 30). The target regions that satisfied the Z-score greater than the 95th percentile plus twice-times absolute standard deviation of the normal control group were retained, and these Z-score was summed as the CNI score [32].

### The relationship of APOB with survival, immune cell infiltration, and drug-gene interaction

To evaluate the association between the expression of *APOB*, *TBC1D32*, and *XAB2* and relapse-free survival (RFS) in breast cancer patients, we utilized the Kaplan-Meier plotter (KMPlot) (http://kmplot.cm/analysis) database. Using the RNA-seq module with these genes as keywords and default settings for other conditions, we analyzed the RFS curves. Subsequently, we focused on *APOB* and employed the Tumor Immune Estimation Resource (TIMER) 2.0 (http://timer.cistrome.org/) database to explore the relationship between gene mutations and immune cell infiltration across different cancer types [33]. By utilizing the Immune Association module followed by the Mutation module, we examined the impact of *APOB* on immune cell infiltration. Finally, we leveraged the Drug-Gene Interaction database (DGIdb) (https://www.dgidb.org/) for the analysis of drug-gene interactions. DGIdb is a comprehensive web resource that consolidates diverse data sources pertaining to drug-gene interactions and gene druggability [34]. We searched for existing drugs or compounds related to *APOB*.

## Results

### Basic information of the samples included in the analysis

Three patients were excluded from the analysis due to either a lack of paracancer samples or the non-compliance of paracancer samples with quality control standards. Tumor samples from eligible patients were subsequently re-arranged and assigned numbers based on their resistance to Herceptin and TKI. The baseline characteristics of all tumor samples are summarized in Supplementary Table S1 for Herceptin sensitivity, and Supplementary Table S2 for TKI sensitivity. Supplementary Table S1 includes sample ID, age, clinical classification, and biomarker information. Supplementary Table S2 includes sample ID, age, clinical classification, and biomarker information. The average age of the enrolled patients was 48 years old, which is relatively young due to the study’s inclusion criteria (< 72 years old) and the early onset of breast cancer in Chinese women (with the peak age being around 50 years old). Samples 1–6, which displayed sensitivity to Herceptin, were selected to form the HE group. Samples 7–12, which exhibited insensitivity to Herceptin, were selected to form the HI group. Samples 13 and 14, both not responsive to Herceptin, were selected as the verification group, referred to as Group YI. Additionally, samples 5–10, 13, and 14, which showed sensitivity to TKI, were chosen to form the TE group, while samples 1, 2, 11, 12, and 15, which displayed insensitivity to TKI, were selected to form the TI group.

### He vs hi

**Fig. 1:**
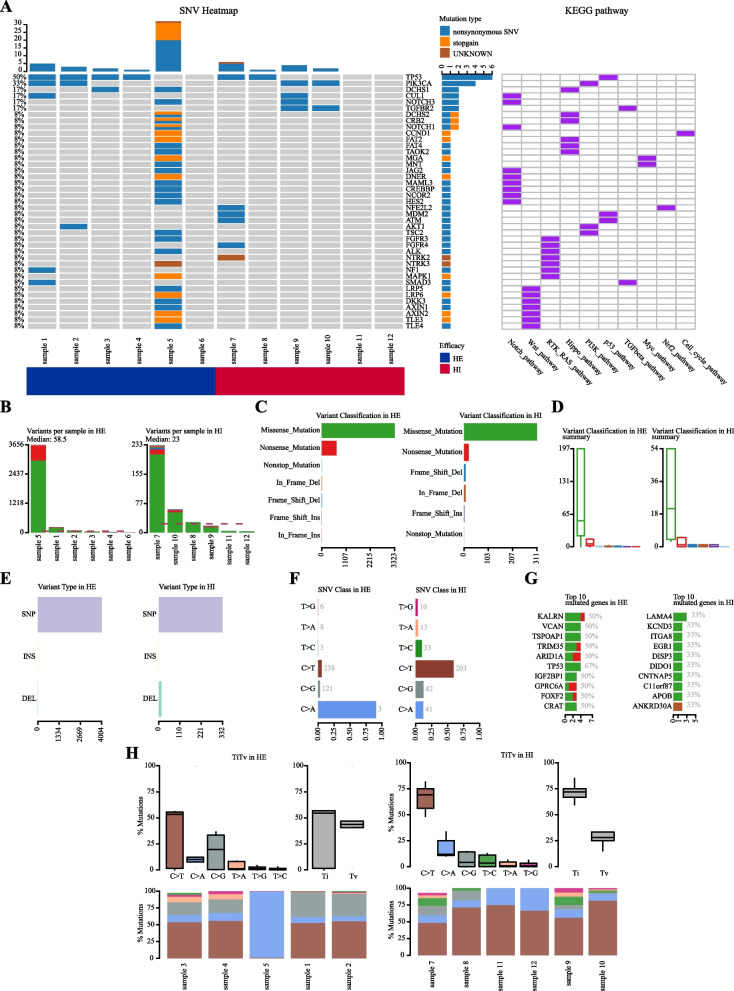
The overall mutational analysis of HE and HI. **A** The overall mutational landscape of HE and HI. **B-G** Type analysis of mutated genes of HE and HI. **H** Proportion of mutations of HE and HI

The most frequently mutated signal pathways in the HE group were Notch, Wnt, Hippo, RTK_RAS, PI3K, Myc, TGFbeta, p53, and Cell_cycle (Fig. 1A). In the HI group, the most commonly mutated signal pathways were p53, Notch, RTK_RAS, TGFbeta, Nrf2, and PI3K (Fig. 1A). Among the HE group samples, sample 5 exhibited the largest difference and the highest number of variants, while sample 7 showed the same characteristics in the HI group (Fig. 1B). Missense mutation was the most common variant classification, and single nucleotide polymorphism (SNP) was the most common variant type in both groups (Fig. 1C-E). However, the types of SNVs differed between the HE group and the HI group. The HE group had the highest frequency of C > A mutations, while the HI group had the highest frequency of C > T mutations (Fig. 1F).

The top 10 mutated genes in the HE group were *KALRN*, *VCAN*, *TSPOAP1*, *TRIM35*, *ARID1A*, *IGF2BP1*, *GPRC6A*, *FOXF2*, *CRAT*, and *TP53*. On the other hand, *LAMA4*, *KCND3*, *ITGA8*, *EGR1*, *DISP3*, *DIDO1*, *CNTNAP5*, *C11orf87*, *APOB*, and *ANKRD30A* were the top 10 mutated genes in the HI group (Fig. 1G). Additionally, there were significant differences in TiTv in the HI group, which may be related to drug resistance (Fig. 1H).

**Fig. 2:**
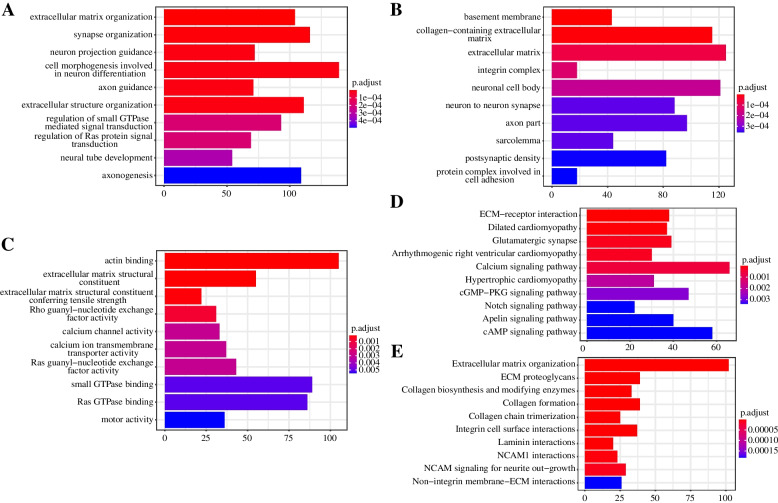
The enrichment analysis of HE and HI. **A** GO-BP analysis of mutated genes; **B** GO-CC analysis of mutated genes; **C** GO-MF analysis of mutated genes; **D** KEGG pathway analysis of mutated genes; **E** reactome pathway analysis of mutated genes

Subsequently, we performed a series of enrichment analyses on the patients’ genes. The result indicated that axon guidance, cell morphogenesis involved in neuron differentiation, extracellular matrix organization, neuron projection guidance and synapse organization are the most enriched GO-BP (Fig. 2A). Basement membrane, collagen−containing extracellular matrix, extracellular matrix, integrin complex, neuronal cell body are the most enriched GO-CC (Fig. 2B). Actin binding, calcium channel activity, extracellular matrix structural constituent, extracellular matrix structural constituent conferring tensile strength, Rho guanyl−nucleotide exchange factor activity are the most enriched GO-MF (Fig. 2C). Among the KEGG pathways, arrhythmogenic right ventricular cardiomyopathy, calcium signaling pathway, dilated cardiomyopathy, ECM-receptor interaction, and glutamatergic synapse were the most enriched (Fig. 2D). Regarding Reactome pathways, the most enriched categories included extracellular matrix organization, ECM proteoglycans, collagen biosynthesis and modifying enzymes, collagen formation, and collagen chain trimerization (Fig. 2E).

**Fig. 3:**
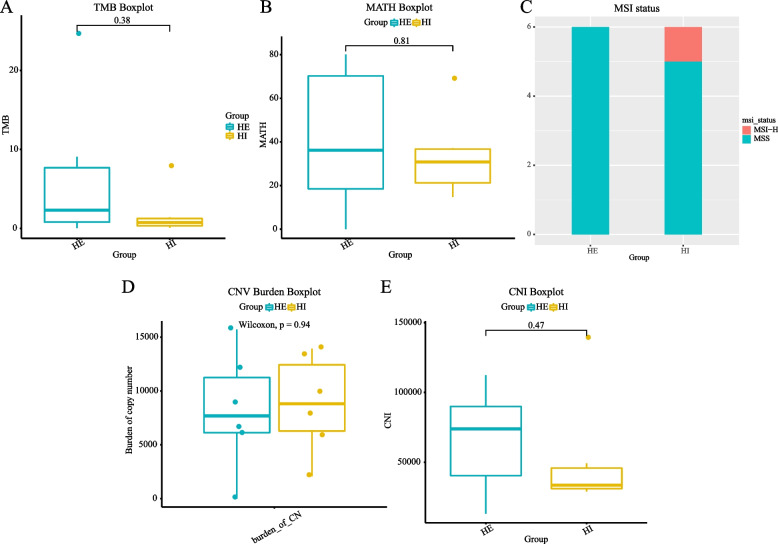
Other WES data of HE and HI. **A** TMB levels of HE and HI; **B** MATH levels of HE and HI; **C** MSI status of HE and HI; **D** CNV burden of HE and HI; **E** CNI levels of HE and HI

The TMB in the HE group was higher than that in the HI group (Fig. 3A). The MATH also tended to be higher in the HE group compared to the HI group (Fig. 3B). There was one patient with MSI-H in the HI group (Fig. 3C). CNV tended to be higher in the HE group compared to the HI group (Fig. 3D), and CNI also tended to be higher in the HE group (Fig. 3E).

### Verification of the insensitive rule of Herceptin

As previously discussed, the identification of differences between the HE and HI groups through WES analysis is of great significance. In order to further investigate the general pattern of drug resistance, we specifically selected samples 13 and 14, which exhibited resistance to Herceptin, in order to establish the YI group. This group was then compared with both the HE and HI groups, with the aim of discerning a comprehensive rule governing drug resistance.

**Fig. 4:**
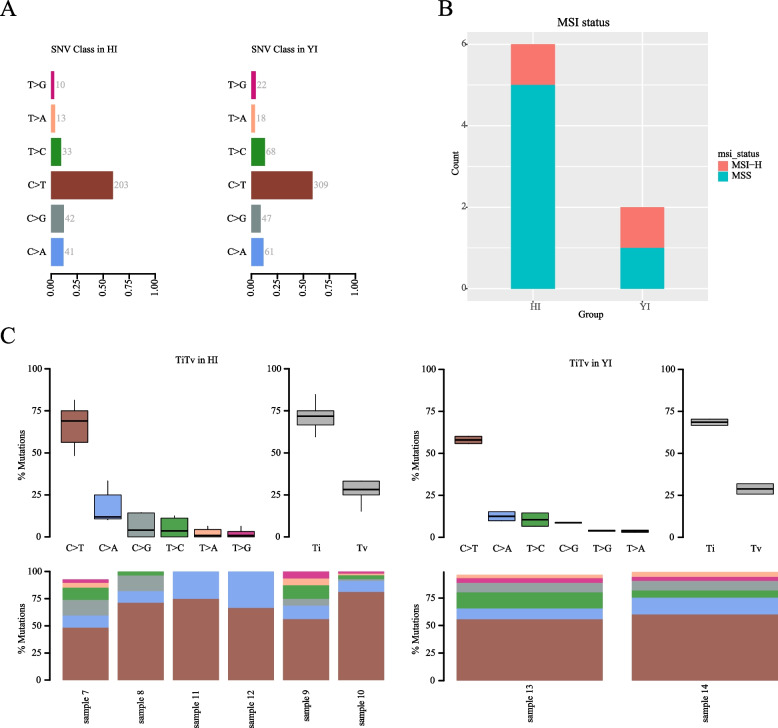
The confirmatory comparison of HI and YI. **A** Type of base mutation of HI and YI. **B** MSI status of YI and HI; **C** Proportion of mutations of YI and HI

The results revealed that the aforementioned differences observed in the HE and HI groups were also evident within the YI group. Notably, the prevalence of C > T mutations was found to be the highest in both the HI and YI groups, in contrast to the HE group (Fig. 4A). While there were no instances of MSI-H in the HE group, both the HI and YI groups had one patient each with this particular genetic anomaly (Fig. 4B). Moreover, in the YI group, there were significant differences observed in TiTV. Similar observations were made in the HI group (Fig. 4C). However, in the HE group, no significant difference was found in TiTV.

### TE vs TI

In an attempt to uncover the patterns associated with TKI resistance in breast cancer patients, we expanded our analysis to include the TE and TI groups using WES. Interestingly, we identified similar patterns of resistance to Herceptin in patients resistant to TKIs.

**Fig. 5:**
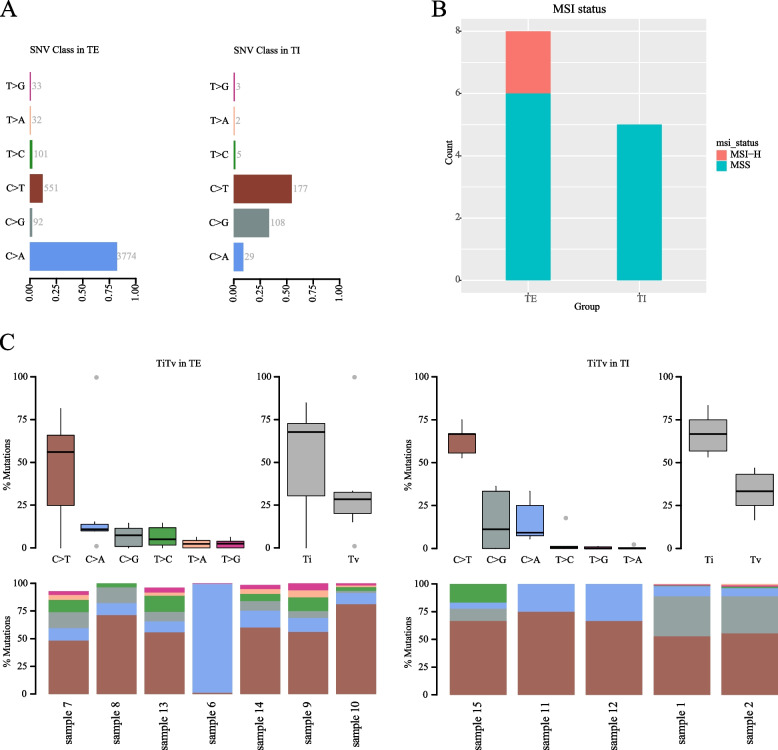
The comparison of TE and TI. **A** Type of base mutation of TE and TI. **B** MSI status of TE and TI; **C** Proportion of mutations of TE and TI

Our findings revealed notable discrepancies in the mutation spectrum between the TE and TI groups. Specifically, we observed a higher frequency of C > T mutations in the TI group compared to the TE group, where C > A mutations prevailed (Fig. 5A).

Furthermore, an intriguing observation was made regarding MSI status. None of the patients in the TI group exhibited MSI-H, while two patients in the TE group displayed MSI-H characteristics (Fig. 5B). This discrepancy may imply distinct molecular pathways driving TKI and Herceptin resistance.

Additionally, in the TI group, there were significant differences observed in TiTV (Fig. 5C). However, in the TE group, no significant difference was found in TiTV. These variations further emphasize unique molecular signatures associated with TKI resistance and reinforce the importance of investigating individualized treatment strategies.

For a comprehensive overview of our WES results, refer to Supplementary Fig. S3–5. These supplementary findings provide additional valuable insights into the genetic landscape of TKI resistance in breast cancer patients.

### Patients sensitive to immunotherapy

**Fig. 6:**
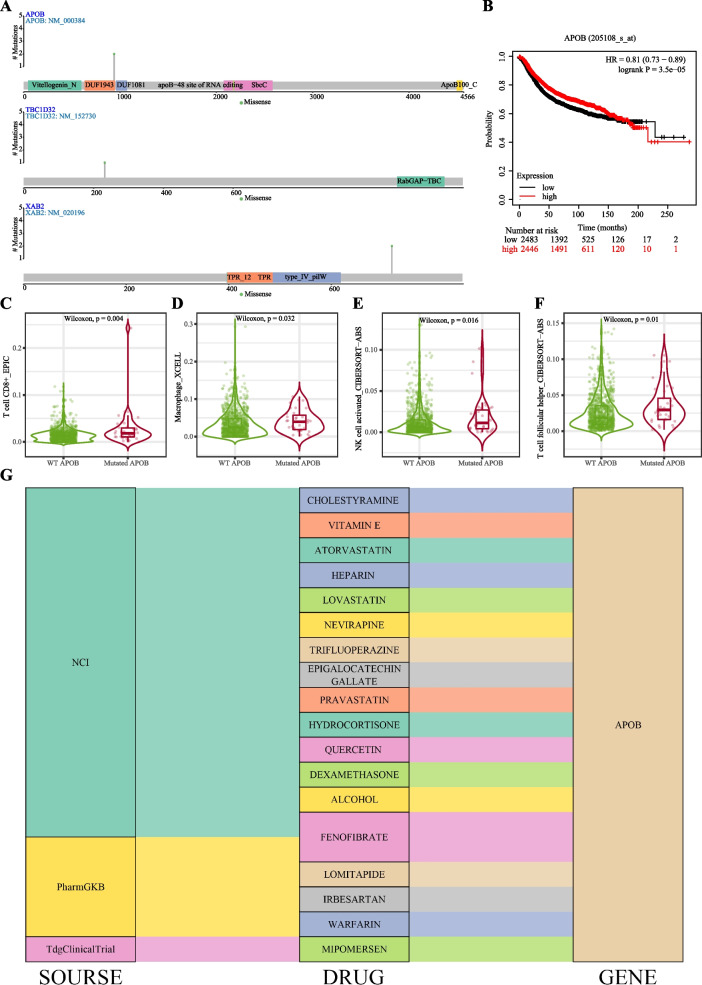
Patients who were insensitive to both Herceptin and TKI but sensitive to immunotherapy were analyzed separately. **A** The gene mutation landscape of APOB, TBC1D32, and XAB2; **B** Kaplan-Meier survival analysis of APOB; **C**-**F** Immune infiltration level of CD8+ T cell, Macrophage, NK cell activated, and T cell follicular helper cell between WT APOB and Mutated APOB in patients with breast cancer. **G** Drug-gene interactions. 18 drugs were obtained that had potential therapeutic effects for targeting APOB

We identified a HER2+ breast cancer patient who displayed resistance to Herceptin and TKI treatment, resulting in relapse. However, intriguingly, this patient exhibited sensitivity to immunotherapy. To investigate the underlying factors contributing to this discrepancy, we conducted detailed analyses of samples 11 and 12 collected from the patient. WES analysis of the patient’s breast cancer sample revealed three unique nonsynonymous SNVs in the *APOB*, *TBC1D32*, and *XAB2* genes. To determine the clinical significance of these genes, we examined their expression levels in a large cohort of breast cancer patients using the KMPlot database (http://kmplot.cm/analysis). As illustrated in Fig. 6, higher expression of *APOB* was associated with worse prognosis. While elevated expression of *TBC1D32* and *XAB32* were not correlated with worse prognosis. Motivated by the patient’s sensitivity to immunotherapy, we sought to explore the potential involvement of immune cells and endothelial cells in mediating this response. Leveraging the TIMER 2.0 database (http://timer.cistrome.org/), we discovered that *APOB* gene mutations influenced the infiltration levels of several immune cell types in breast cancer. Specifically, breast cancer patients with APOB mutations exhibited higher infiltration levels of CD8 + T cell, Macrophage, activated NK cell, and T follicular helper cells, with corresponding log2 fold changes of 0.915524732, 0.564052222, 0.738113493, and 0.446620369, respectively (Fig. 6C-F).

Therefore, we propose *APOB* as a promising therapeutic target and aim to investigate potential drugs that may exert therapeutic effects. Utilizing the DGIdb database (https://www.dgidb.org/), we identified 18 drugs with potential interactions with APOB (Fig. 6G). However, further research is warranted to validate the efficacy of these drugs.

## Discussion

HER2, a transmembrane tyrosine kinase receptor, plays a crucial role in various cellular processes such as proliferation, survival, differentiation, angiogenesis, invasion, and metastasis [35]. It belongs to the epidermal growth factor receptor (EGFR) family, which consists of EGFR/HER1, HER2, HER3, and HER4 [36]. HER2-positive breast cancer is associated with a poor prognosis; however, targeted therapy has shown promising results for this subtype of breast cancer [5]. Notably, Herceptin was the first approved drug specifically designed to target HER2 and has demonstrated efficacy in clinical trials [9, 10]. Lapatinib, the first TKI approved for breast cancer, targets both EGFR and HER2. In 2007, the combination of lapatinib and capecitabine was approved by the Federal Drug Administration (FDA) as a second-line therapy for advanced breast cancer [11]. Neratinib, a second-generation TKI, irreversibly inhibits multiple tyrosine kinase receptors [12]. Studies have shown that compared to Herceptin, neratinib can significantly reduce the incidence of central nervous system recurrence and delay central nervous system metastasis [13]. Tucatinib, another TKI, is a selective inhibitor of HER2 and has demonstrated superiority over lapatinib or neratinib in mice with HER2+ intracranial xenografts [14]. However, despite the success of these targeted therapies, more than 50% of patients either do not respond or develop resistance to Herceptin [16–18]. Similar challenges are observed in patients treated with TKIs. Numerous experiments have been conducted to elucidate the mechanisms underlying the lack of response or the development of resistance to TKIs [19–21]. Nevertheless, the exact mechanisms remain unclear.

In this study, we divided the patients into different groups for comparison and validation purposes. The HE group (samples 1–6) was compared to the HI group (samples 7–12), while the YI group (samples 13 and 14) served as a validation group. Our findings revealed several important observations. Firstly, we observed a higher percentage of C > T mutations in the resistant groups (HI and YI). This suggests that C > T mutations may be associated with resistance to Herceptin. Additionally, significant differences in TiTv and MSI-H status were observed in the resistant groups. These findings indicated that a higher percentage of C > T mutations, significant differences in TiTv, and MSI-H status may suggest resistance to Herceptin. It is of significance that these rules may guide clinicians to timely detect Herceptin resistance before or during treatment to adjust medication.

Similarly, comparing the TE group (samples 5–10, 13, and 14) with the TI group (samples 1, 2, 11, 12, and 15) revealed comparable results, suggesting that higher C > T mutation rates, significant differences in TiTv, and without MSI-H could indicate TKI resistance in breast cancer patients. It is important that these rules may guide clinicians to timely detect TKI resistance before or during treatment to adjust medication.

As mutant spectrum analysis found that the more malignant patients with lung cancer had higher percentage of C > T and significant difference in TiTv, while common patients with lung cancer had the more conversion from C to A [37]. Additionally, it has been reported that patients with lung cancer who exhibit decreased sensitivity to TKIs have a higher TiTv ratio compared to other patients [38]. Generally, cancers with more aggressive characteristics tend to be more resistant to drugs. Therefore, based our results that higher percentage of C > T and significant difference in TiTv, we speculate that these molecular alterations may have important implications in reflecting malignant behaviors and drug resistance in cancer patients. Notably, such phenomena have rarely been reported in breast cancer, making these results particularly valuable in guiding drug selection for breast cancer patients, particularly those with HER2+ status. This could greatly facilitate decision-making and save time in clinical practice.

In recent years, most of the research focus on MSI-H cancer patients is on the response to PD-1 inhibitors. This links somatic hypermutation and new epitope formation to immunotherapy responses and relies on a simple, widely used diagnostic test [39]. In May 2017, the FDA accelerated approval of the PD-1 inhibitor pembrolizumab (Keytruda) for adults and children with unresectable or metastatic MSI-H solid tumors [40]. However, associations between MSI and Herceptin resistance or TKI resistance are rarely reported. In our study, we found that breast cancer patients with MSI-H may tend to be Herceptin insensitive, breast cancer patients without MSI-H may tend to be TKI insensitive. It will broaden the implication of estimation of MSI status, and contribute to discover the deeper mechanism of drug resistance and further benefit the patients of breast cancer.

Interestingly, we also analyzed a patient who exhibited resistance to both Herceptin and TKI treatments but responded well to immunotherapy. We found that mutated *APOB* may function during the resistance to Herceptin and TKI. Previous studies have shown that APOB-lipoproteins and their components are involved in regulating intracellular metabolism and are associated with various tumor-related phenotypes such as proliferation, anchorage-independent growth, epithelial-mesenchymal transition, and cancer invasion [41]. For instance, APOB has been identified as a risk factor in breast cancer and has been implicated in promoting the risk of intraocular metastasis in breast cancer patients [42]. These findings provide additional support for considering mutated *APOB* as a detrimental factor during Herceptin and TKI treatment.

On the other hand, mutated *APOB* has been found to be associated with immune infiltration in hepatocellular cancer [43]. However, the relationship between mutated *APOB*, immune infiltration, and response to immunotherapy in breast cancer remains poorly characterized. In our study, we observed a positive correlation between mutated *APOB* and the levels of immune infiltration of CD8+ T cells, macrophages, activated NK cells, and T follicular helper cells. Increased levels of immune infiltration of CD8+ T cells have been reported to be associated with improved response to immunotherapy in breast cancer patients [44–46]. Mechanistically, intratumoral CD8+ T cells exhibiting a tissue-resident memory phenotype have been shown to mediate local immunity and immune checkpoint responses in breast cancer. Macrophage [47], activated NK cell [48], and T follicular helper cells [49] have also been associated with anti-tumor effects during immunotherapy. Furthermore, T follicular helper cells were demonstrated to restore CD8+ T cell -dependent antitumor immunity and anti-PD-L1/PD-1 efficacy [50]. Therefore, it is plausible that mutated *APOB* may exert its influence by increasing the levels of immune infiltration of these four types of immune cells. Further experimental validation is warranted to confirm these observations. The identification of mutated *APOB* as a potential biomarker for Herceptin and TKI resistance could be beneficial for breast cancer patients, as it would enable the prompt administration of immunotherapy. Additionally, the inclusion of drugs targeting *APOB* in the therapeutic regimen may further enhance treatment efficacy.

Overall, we revealed the genomic difference between patients who were resistant to anti-HER2 drug and patients who were not. And identified Herceptin-resistant rules and TKI-resistant rules. What’s more, we found a new mutated gene as candidate which can predict the response to immunotherapy. While further molecular biology studies to validate the results mentioned above and a larger-scale of samples would be required to confirm our findings.

## Conclusion

In conclusion, somatic mutations in patients with breast cancer were detected by WES technique, annotated their functions using bioinformatics approaches, and further discover immune-features and potential drugs through online tools. The higher percentage of C > T, the significant difference in TiTv, and MSI-H may indicate Herceptin resistance. The higher percentage of C > T, the significant difference in TiTv, and no MSI-H may indicate TKI resistance in the patients. For those patients who are resistant to both Herceptin and TKI, mutated *APOB* may play an important role in resistance. These findings contribute to a better understanding of the molecular pathogenesis of resistance to Herceptin and TKI. It may provide guidance for the selection of therapy for breast cancer. We will collect more clinical samples and validate these findings in the future studies.

### Supplementary Information

**Additional file 1:** **Supplementary Table S1.** Baseline information of the Herceptin-sensitive (HE) group and the Herceptin-insensitive (HI) group.

**Additional file 2:** **Supplementary Table S2.** Baseline information of the TKI-sensitive (TE) group and the TKI-insensitive (TI) group.

**Additional file 3:** **Supplementary Figure S3.** Type analysis of mutated genes of TE and TI.

**Additional file 4:** **Supplementary Figure S4.** The enrichment analysis of TE and TI. (A) GO-BP analysis of mutated genes; (B) GO-CC analysis of mutated genes; (C) GO-MF analysis of mutated genes; (D) KEGG pathway analysis of mutated genes; (E) reactome pathway analysis of mutated genes.

**Additional file 5:** **Supplementary Figure S5.** Other WES data of TE and TI. (A) TMB levels of TE and TI; (B) MATH levels of TE and TI; (C) CNV burden of TE and TI; (D) CNI levels of TE and TI.

## Data Availability

The raw sequence data reported in this paper have been deposited in the Genome Sequence Archive (Genomics, Proteomics & Bioinformatics 2021) in National Genomics Data Center (Nucleic Acids Res 2022), China National Center for Bioinformation / Beijing Institute of Genomics, Chinese Academy of Sciences (GSA-Human: HRA004623) that are publicly accessible at https://ngdc.cncb.ac.cn/gsa-human.

## Abbreviations

WES: Whole-exome sequencing
TKIs: Tyrosine kinase inhibitors
GATK: Genome Analysis Toolkit
HI: Herceptin-insensitive
HE: Herceptin-sensitive
MSI: Microsatellite instability
TiTv: Transition-transversion
TI: TKI- insensitive
TE: TKI-sensitive
SNVs: Single nucleotide substitutions
GO: Gene ontology
BP: Biological process
MF: Molecular function
CC: Cellular component
KEGG: Kyoto Encyclopedia of Genes and Genomes
TMB: Tumor mutational burden
MATH: Mutant allele tumor heterogeneity
STR: Short tandem repeat
SD: Standard deviation
CNV: Copy number variation
CNI: Copy number instabilit
RFS: Relapse-free survival
KMPlot: Kaplan-Meier plotter
TIMER: Tumor Immune Estimation Resource
DGIdb: Drug-Gene Interaction database
SNP: Single nucleotide polymorphism
EGFR: Epidermal growth factor receptor
FDA: Federal Drug Administration
