# Supplementary material for: Based on whole-exome sequencing to explore the rule of Herceptin and TKI resistance in breast cancer patients
Source: BMC Med Genomics. 2024 Jan 19;17:25. doi: 10.1186/s12920-023-01762-x (PMC10799408; doi:10.1186/s12920-023-01762-x)
Supplement: Supplementary file 2 — Additional file 2: Supplementary Table S2. Baseline information of the TKI-sensitive (TE) group and the TKI-insensitive (TI) group. [file 12920_2023_1762_MOESM2_ESM.docx]

Supplementary Table S2. Baseline information of the TKI-sensitive (TE) group and the TKI-insensitive (TI) group.

| Sample ID | TKI-sensitive | Age | Clinical classification | ER | PR | HER2 | Ki67 |
| --- | --- | --- | --- | --- | --- | --- | --- |
| Sample 5 | Yes | 31 | (left) Invasive breast carcinoma, non-specific type (invasive ductal carcinoma), grade 3 | (-) | (-) | (3+) | (+, 40%) |
| Sample 6 | Yes | 31 | (left) Invasive breast carcinoma, non-specific type (invasive ductal carcinoma), grade 3 | (-) | (-), | (3+) | (+, 40%) |
| Sample 7 | Yes | 46 | (right) Invasive carcinoma of the breast, non-specific type (invasive ductal carcinoma), grade 3, some micropapillary carcinoma with high-grade ductal carcinoma in situ (acne) | (+, 30%) | (+,10%) | (3+) | (+, 30%) |
| Sample 8 | Yes | 46 | (right) Invasive carcinoma of the breast, non-specific type (invasive ductal carcinoma), grade 3, some micropapillary carcinoma with high-grade ductal carcinoma in situ (acne) | (+, 30%) | (+,10%) | (3+) | (+, 30%) |
| Sample 9 | Yes | 71 | (left) Invasive breast carcinoma, non-specific type (invasive ductal carcinoma), grade 3 | (-) | (-) | (3+) | (+, 40%) |
| Sample 10 | Yes | 71 | (left) Invasive breast carcinoma, non-specific type (invasive ductal carcinoma), grade 3 | (-) | (-) | (3+) | (+, 40%) |
| Sample 13 | Yes | 54 | (right) Invasive carcinoma of the breast, non-specific type (invasive ductal carcinoma), grade 3, with high-grade ductal carcinoma in situ (acne) | (-) | (-) | (3+) | (+, 20%) |
| Sample 14 | Yes | 65 | (right) Invasive carcinoma of the breast, non-specific type (invasive ductal carcinoma), grade 3 | (-) | (-) | (2+) | (+, 20%) |
| Sample 1 | No | 53 | (left) Invasive breast carcinoma, non-specific type (invasive ductal carcinoma), grade 3 | (+++, 70%) | (+, 10%) | (2+) | (+, 40%) |
| Sample 2 | No | 53 | (left) Invasive breast carcinoma, non-specific type (invasive ductal carcinoma), grade 3 | (+++, 70%) | (+, 10%) | (2+) | (+, 40%) |
| Sample 11 | No | 61 | (left) Invasive carcinoma of the breast, non-specific type (invasive ductal carcinoma), grade 3, some micropapillary carcinoma with partial necrosis | (-) | (-) | (3+) | (+, 70%) |
| Sample 12 | No | 61 | (left) Invasive carcinoma of the breast, non-specific type (invasive ductal carcinoma), grade 3, some micropapillary carcinoma with partial necrosis | (-) | (-) | (3+), | (+, 70%) |
| Sample 15 | No | 49 | (left) Invasive carcinoma of the breast, non-specific type (invasive ductal carcinoma), grade 3, some with micropapillary structures | (+++,80%) | (+,20%) | (3+) | (+, 20%) |
