# Supplementary figures and images for: Based on whole-exome sequencing to explore the rule of Herceptin and TKI resistance in breast cancer patients
Source: BMC Med Genomics. 2024 Jan 19;17:25. doi: 10.1186/s12920-023-01762-x (PMC10799408; doi:10.1186/s12920-023-01762-x)

A

SNV Heatmap

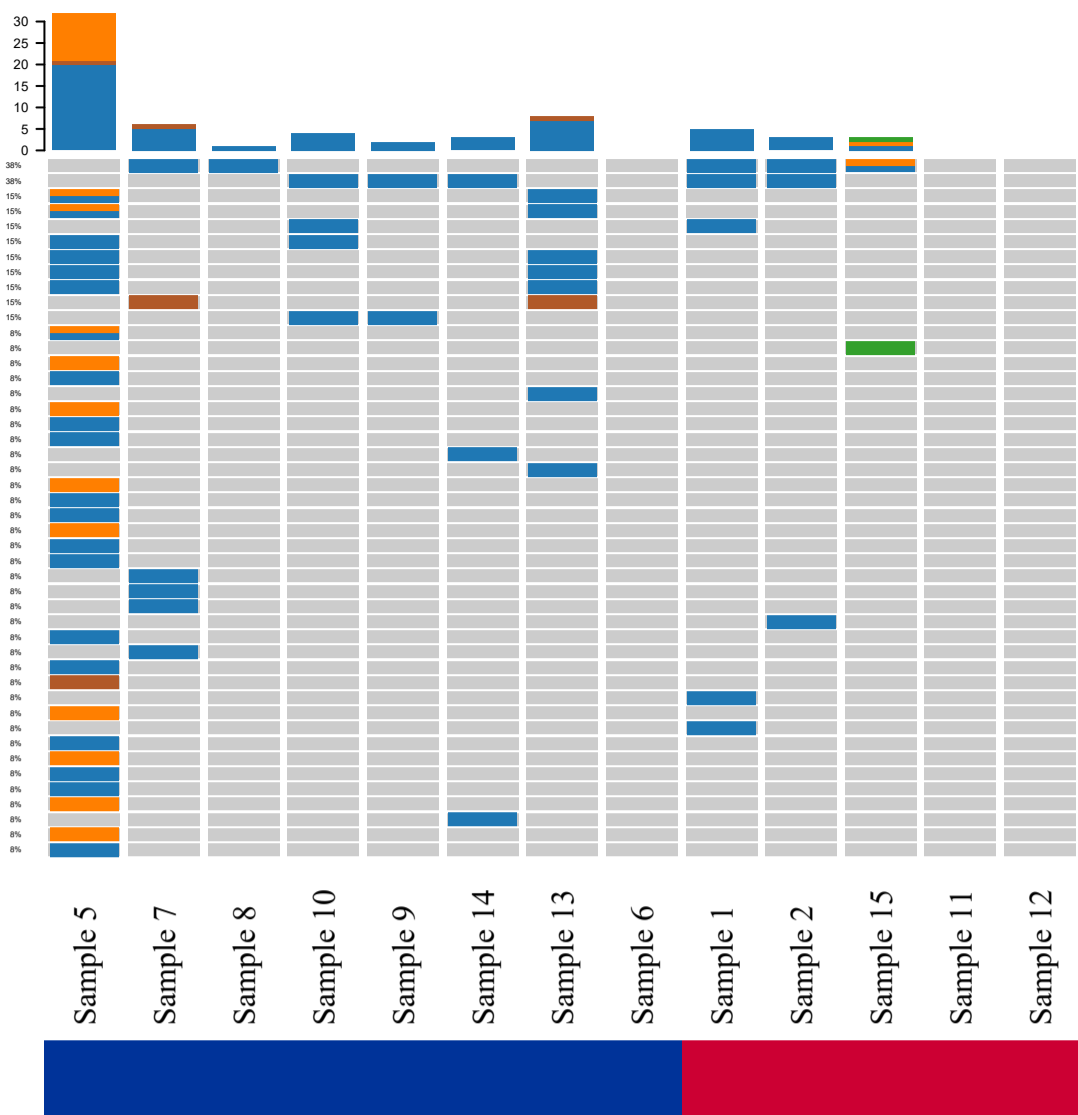

KEGG pathway

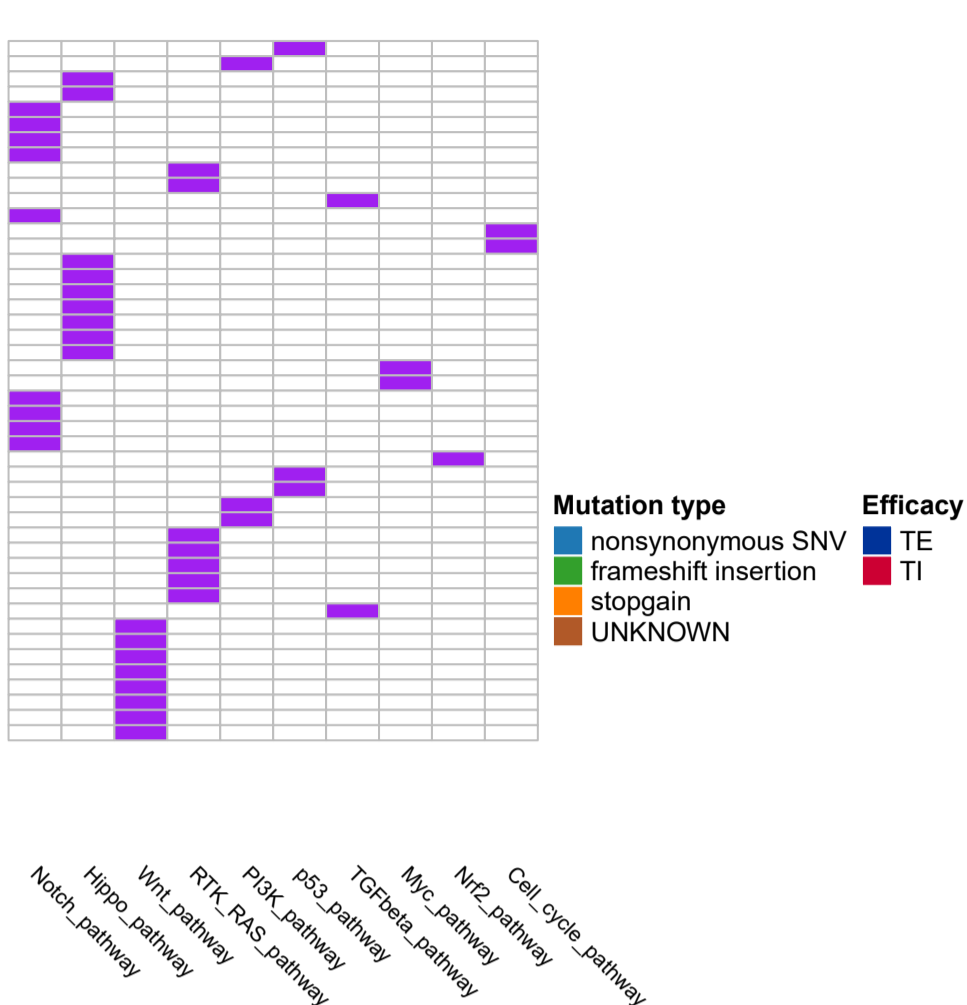

B

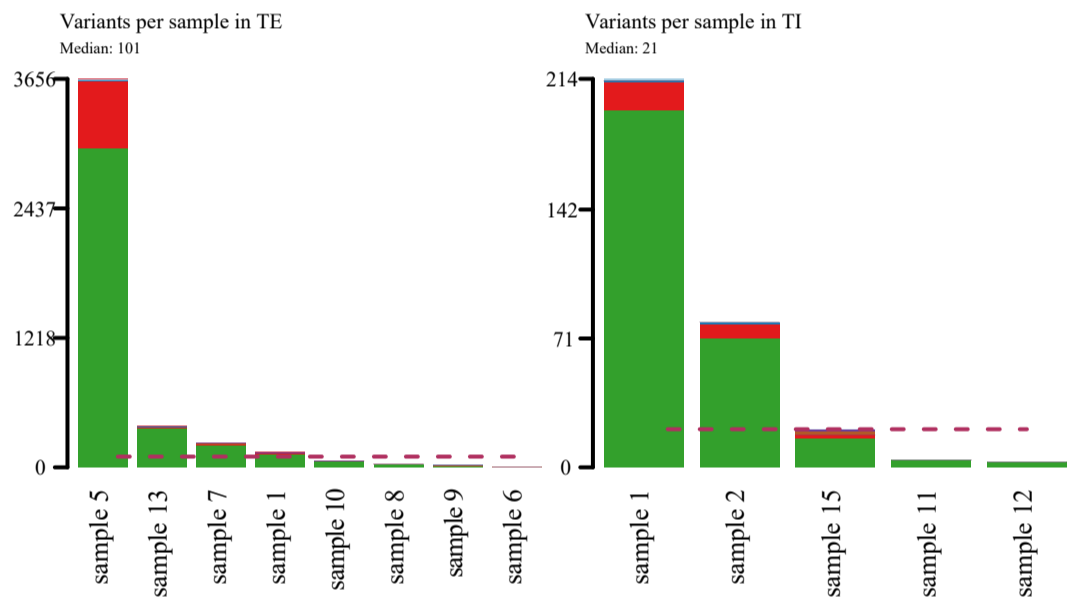

C

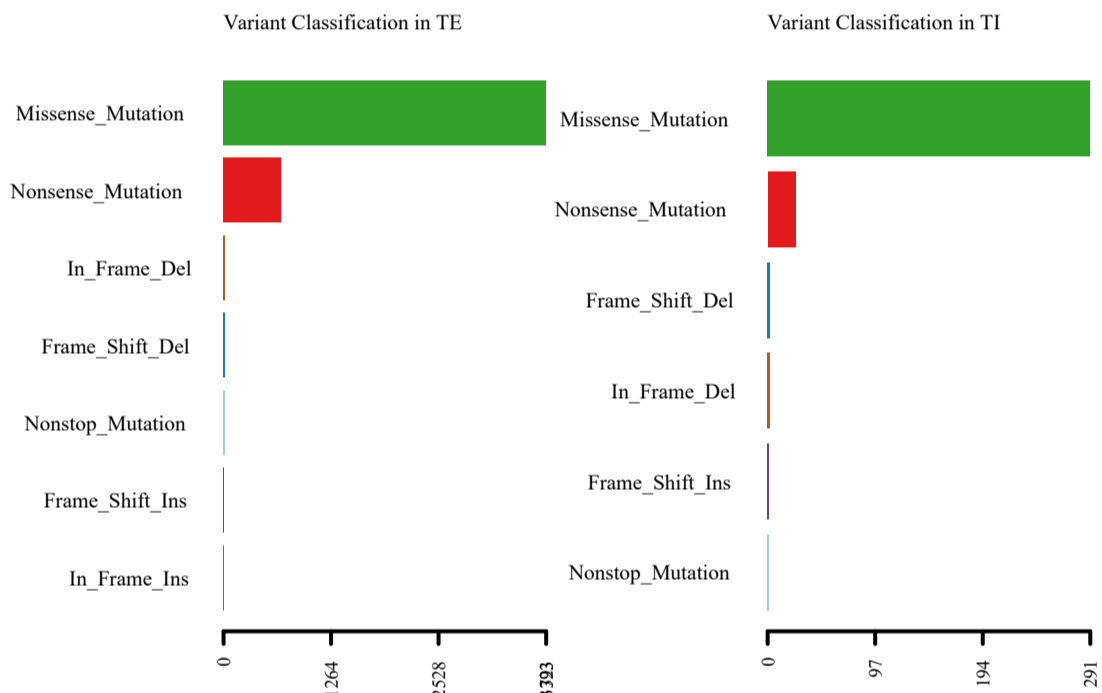

D

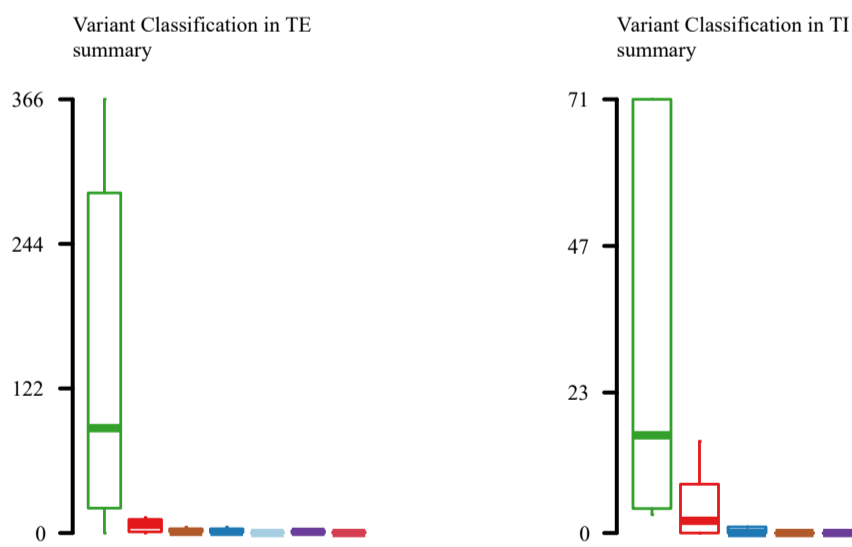

E

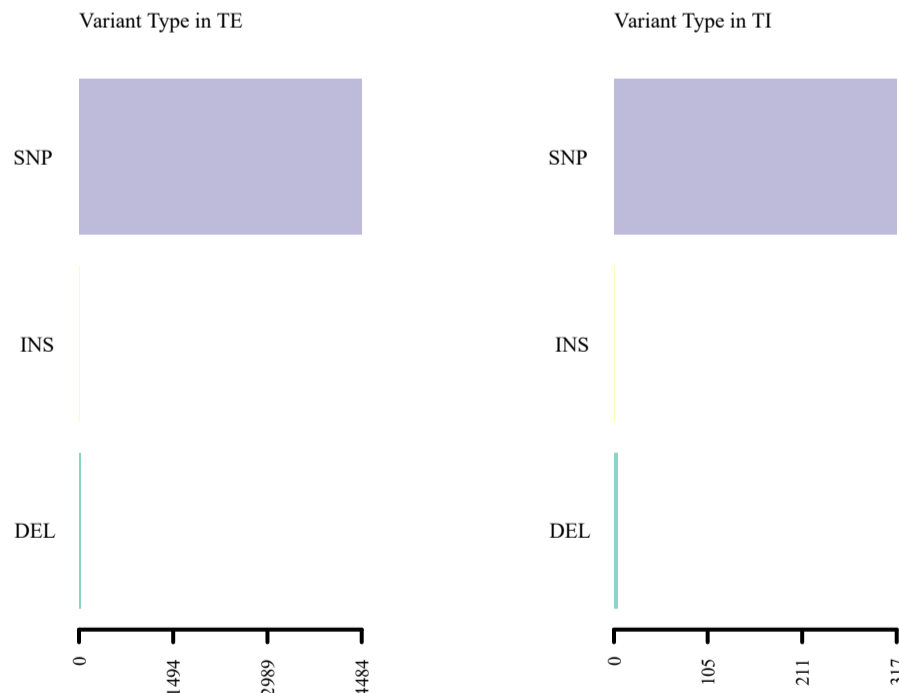

F

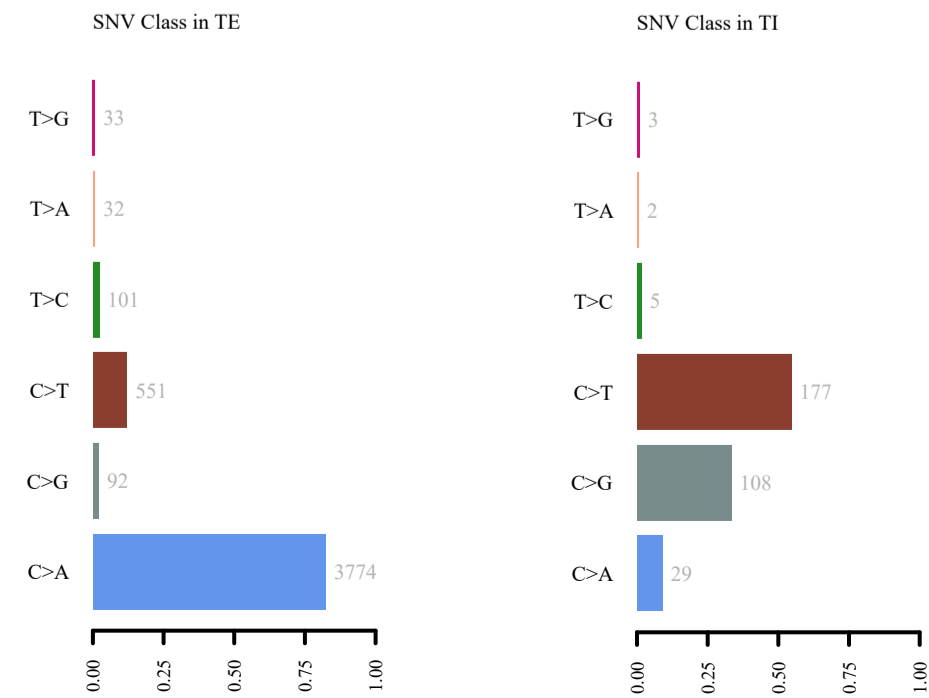

G

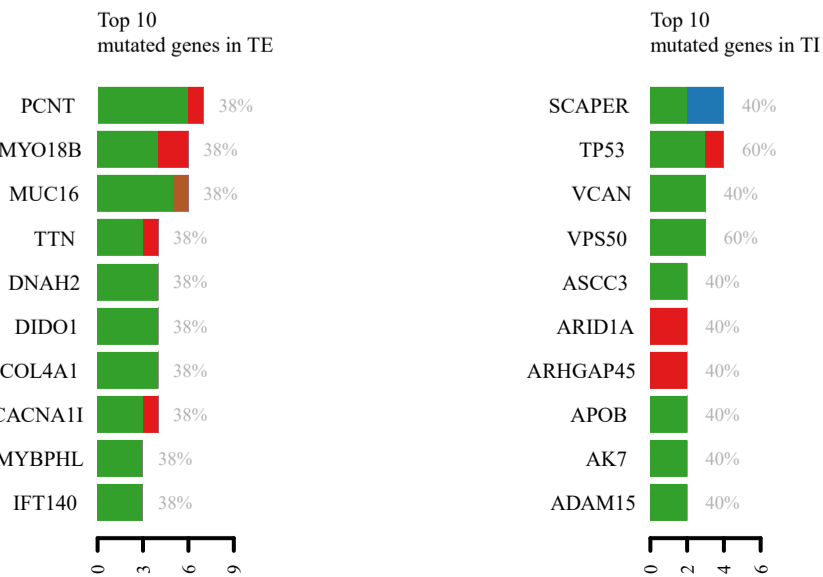

Supplement: Supplementary file 3 — Additional file 3: Supplementary Figure S3. Type analysis of mutated genes of TE and TI. [file 12920_2023_1762_MOESM3_ESM.pdf]

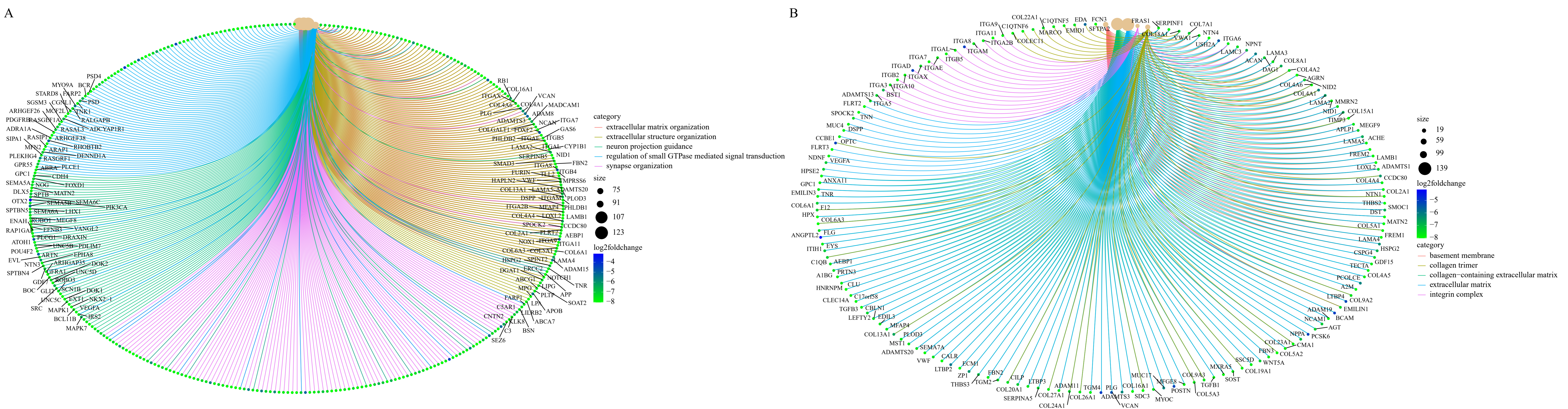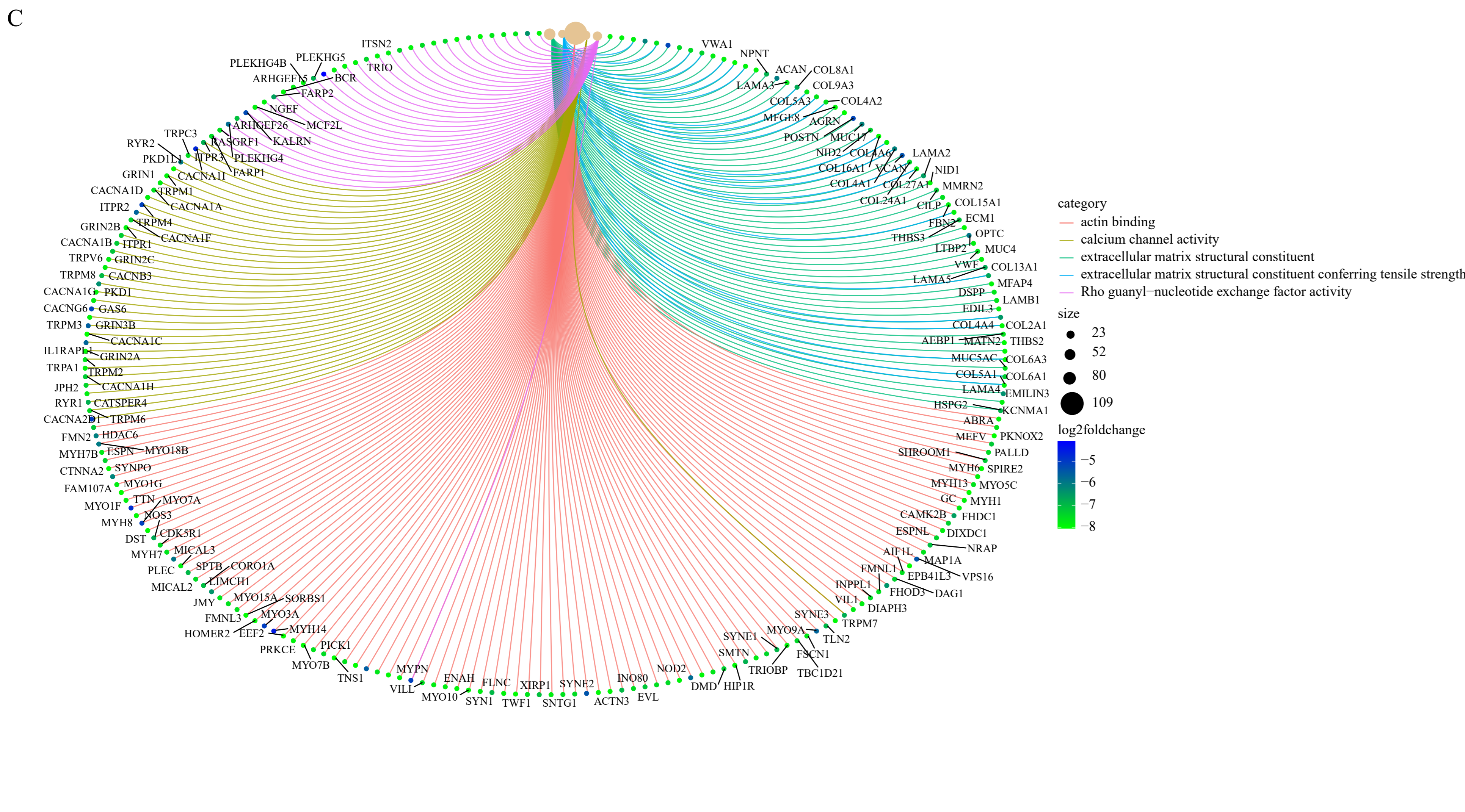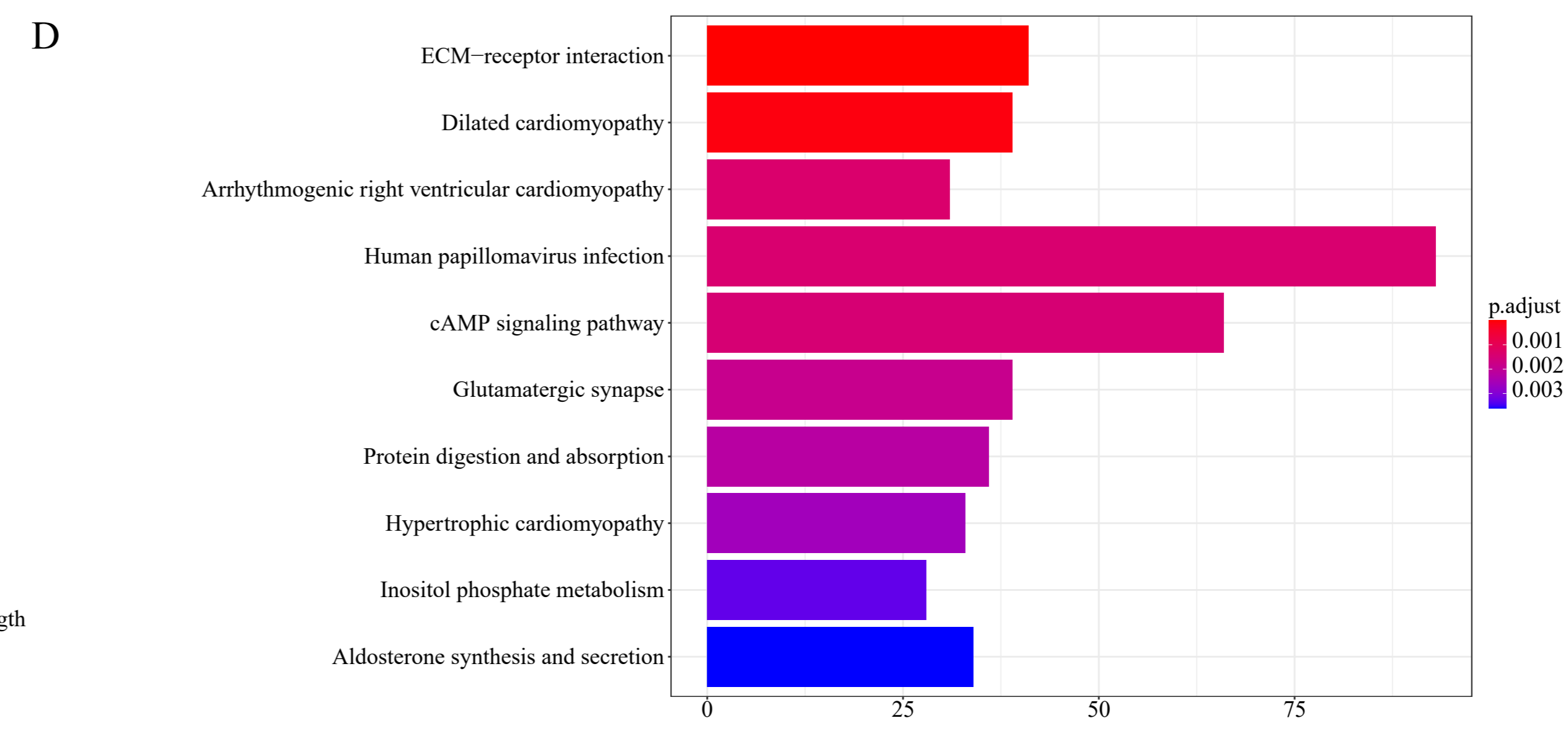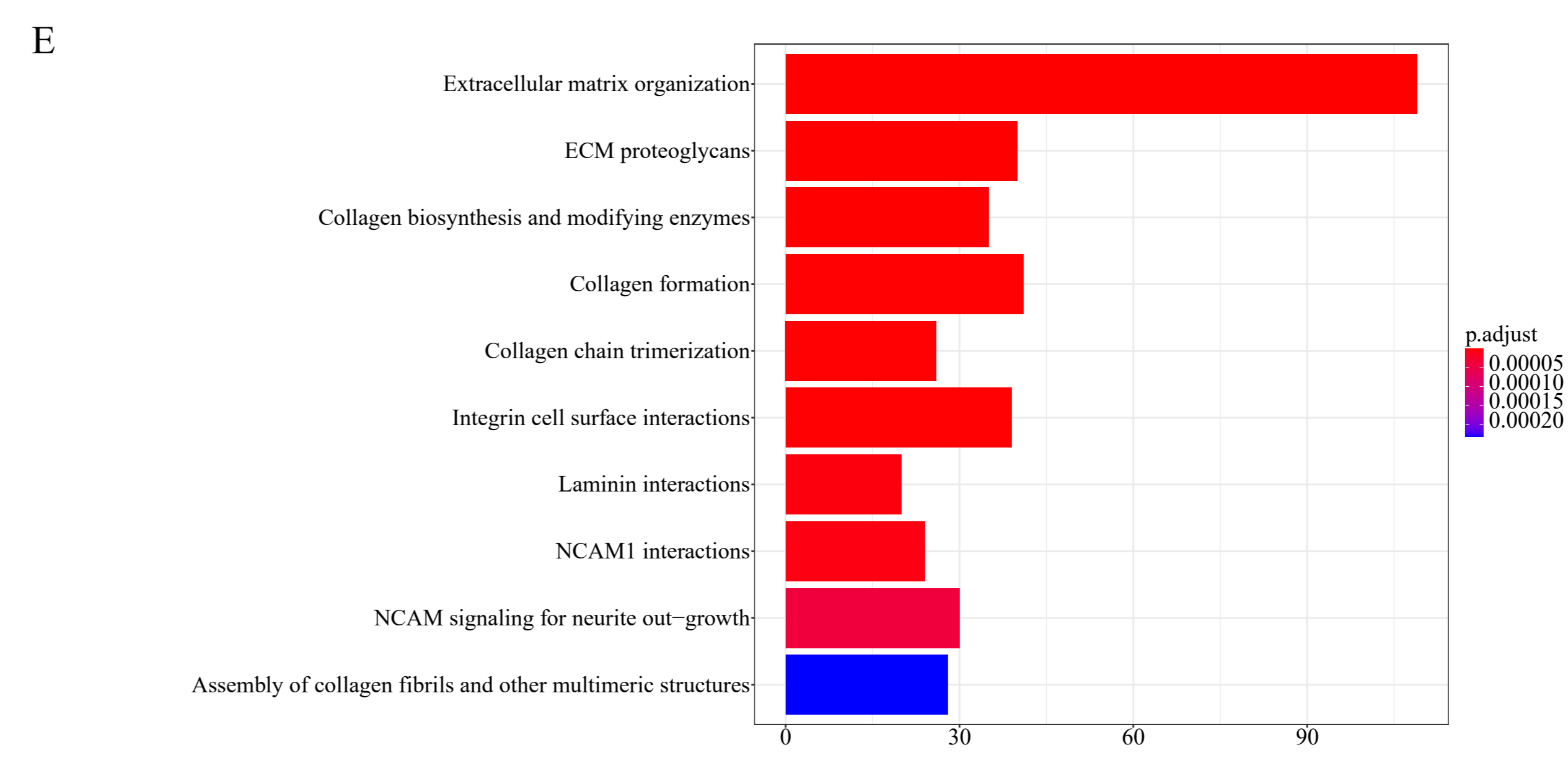

Supplement: Supplementary file 4 — Additional file 4: Supplementary Figure S4. The enrichment analysis of TE and TI. (A) GO-BP analysis of mutated genes; (B) GO-CC analysis of mutated genes; (C) GO-MF analysis of mutated genes; (D) KEGG pathway analysis of mutated genes; (E) reactome pathway analysis of mutated genes. [file 12920_2023_1762_MOESM4_ESM.pdf]

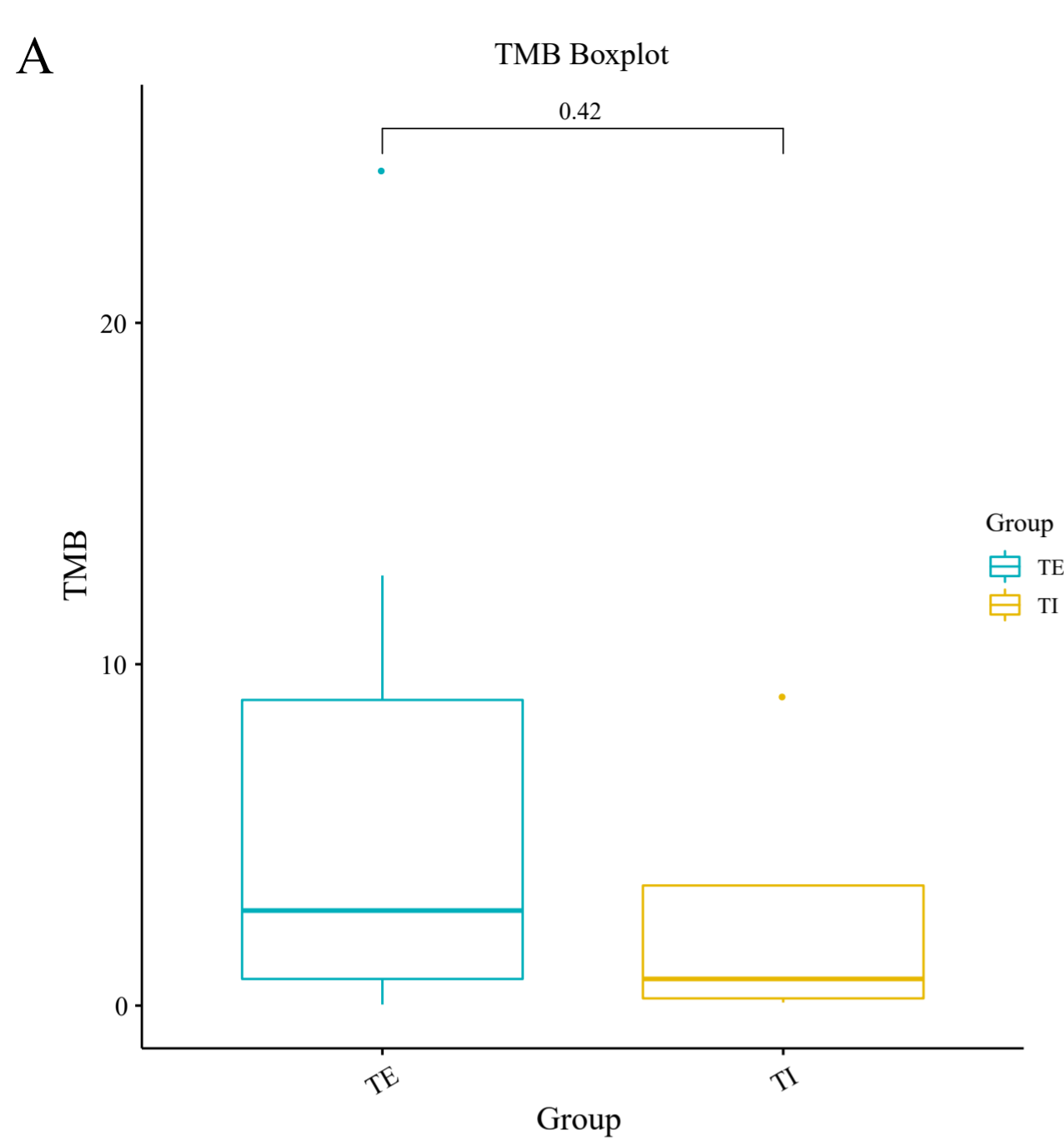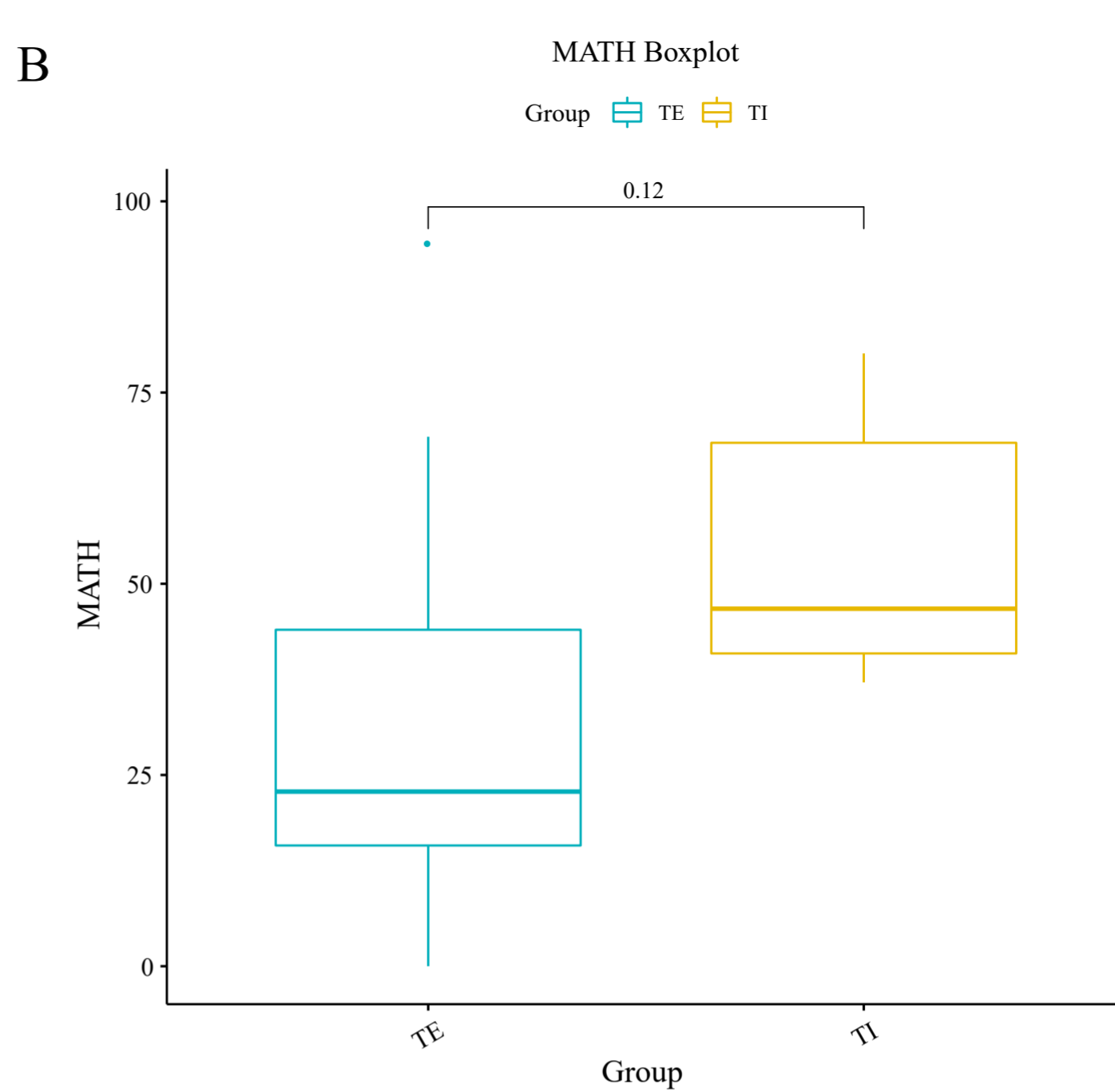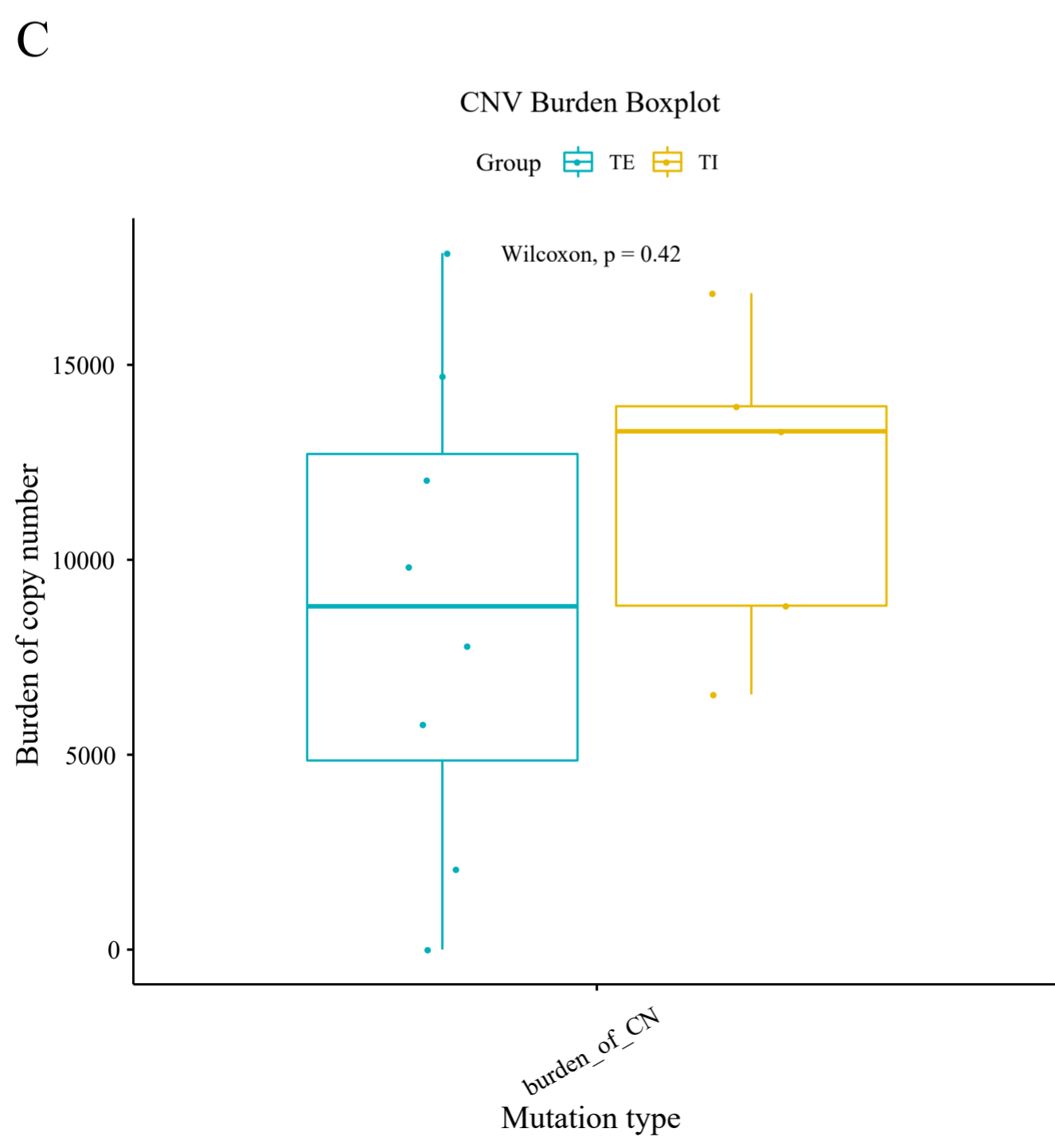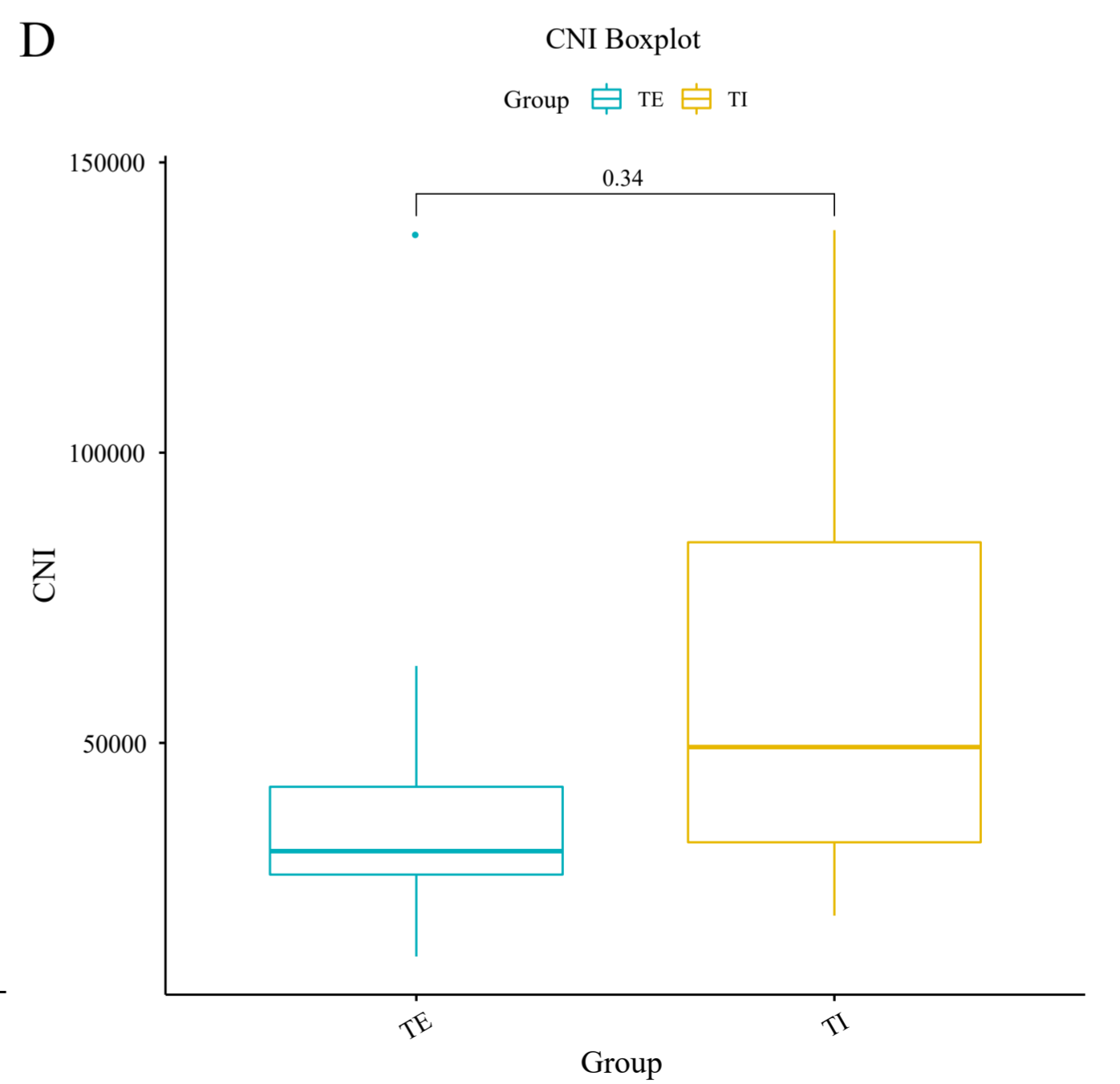

Supplement: Supplementary file 5 — Additional file 5: Supplementary Figure S5. Other WES data of TE and TI. (A) TMB levels of TE and TI; (B) MATH levels of TE and TI; (C) CNV burden of TE and TI; (D) CNI levels of TE and TI. [file 12920_2023_1762_MOESM5_ESM.pdf]
